# Decline in Liver Mitochondria Metabolic Function Is Restored by Hochuekkito Through Sirtuin 1 in Aged Mice With Malnutrition

**DOI:** 10.3389/fphys.2022.848960

**Published:** 2022-03-01

**Authors:** Miwa Nahata, Naoki Fujitsuka, Hitomi Sekine, Chika Shimobori, Katsuya Ohbuchi, Seiichi Iizuka, Sachiko Mogami, Shunsuke Ohnishi, Hiroshi Takeda

**Affiliations:** ^1^Tsumura Kampo Research Laboratories, Tsumura & Co., Ibaraki, Japan; ^2^Tsumura Advanced Technology Research Laboratories, Tsumura & Co., Ibaraki, Japan; ^3^Faculty of Pharmaceutical Sciences, Hokkaido University, Sapporo, Japan; ^4^Gastroenterology, Tokeidai Memorial Hospital, Sapporo, Japan

**Keywords:** aging, hochuekkito, metabolomics, mitochondria, sirtuin1

## Abstract

Malnutrition impairs basic daily activities and leads to physical frailty, which is aggravated in the elderly compared with young adults. It is also well-known that the elderly are more vulnerable to metabolic stress. Therefore, in this study, using a food restricted (FR) mouse, we aimed to evaluate the effect of aging on locomotor activity and liver metabolic function. Further, we also investigated the involvement of hepatic mitochondria in liver metabolic function during aging, as well as the therapeutic benefit of the traditional Japanese medicine, hochuekkito (HET). Our findings indicated that following food restriction provided as 30% of *ad libitum* intake for 5 days, the locomotor activity was lower in 23–26-month-old (aged) mice than in 9-week-old (young) mice. Further, compared with young mice, aged mice exhibited significant decreases in the levels of metabolites related to the urea cycle, mitochondrial function, and anti-oxidative stress. The livers of the aged mice also showed a greater decrease in mitochondrial DNA copy number than young mice. Furthermore, the gene expression levels of sirtuin 1 (SIRT1) and mitochondrial biogenesis-related regulators were attenuated in aged mice. However, these changes were partially restored by HET treatment, which also improved locomotor activity, and combined treatment with alanine resulted in more significant effects in this regard. Therefore, our findings suggested that the decrease in locomotor activity in aged FR mice was associated with a decline in the metabolic function of hepatic mitochondria via decreased SIRT1 expression, which was restored by HET treatment. This implies that enhancing the metabolic function of liver mitochondria can contribute to alleviating energy deficiency in the elderly.

## Introduction

A decline in nutritional intake in the elderly is associated with various physiological, psychological, and social factors, and leads to marked malnutrition (Saka et al., 2010). Further, malnutrition leads to the onset of frailty and sarcopenia, and consequently causes the deterioration of physical and mental functions (Cederholm et al., 2017). Moreover, it is a major risk factor for post-operative outcomes, morbidity, prolonged hospitalization, and mortality among older patients (Xue, 2011; Rose et al., 2021). Thus, it can lead to common health care issues among the elderly. In general, it has been recognized that more adequate nutrition support is required to maintain the body mass of elderly patients given that they are vulnerable to metabolic stress, such as malnutrition compared with young adults (Shizgal et al., 1992); however, the precise mechanisms have not yet been fully elucidated.

The liver, which is the central metabolic organ involved in controlling systemic energy metabolism, molecular biosynthesis, and detoxification, is metabolically connected to the different tissues of the body and provides essential energy via hepatic glucose and lipid homeostasis during fasting and starvation (Hunt et al., 2019). Specifically, hepatic mitochondria have been recognized as major cellular energy generators (Popov, 2020); however, they also simultaneously participate in multifaceted metabolic reactions and also promote cellular adaptation to nutrient deprivation (Spinelli and Haigis, 2018). Further, it has been observed that during aging, oxidative stress induces mitochondrial dysfunction, which is mechanistically linked to several age-related pathologies, such as metabolic diseases, neurodegenerative disorders, cardiovascular diseases, and cancer (Srivastava, 2017; Kumar and Reichert, 2021). Thus, we hypothesized that in elderly patients with malnutrition, metabolic function of hepatic mitochondria is impaired, resulting in physical frailty.

Hochuekkito (HET), which is a commonly used traditional Japanese medicine that contains a powdered product extracted from ten medicinal herbs, has been approved by the Japanese Ministry of Health, Labor, and Welfare for clinical use. Specifically, HET is used for the treatment of physical function failure in patients following disease and surgery. Clinical studies have also demonstrated that HET has beneficial effects on fatigue, nutritional status, chronic wounds, and quality of life in elderly patients with malnutrition (Satoh et al., 2005; Hamada et al., 2018; Akita et al., 2019). Its clinical efficacy has also been supported by studies using animal models (Park et al., 2017; Cai and Yang, 2019; Isago et al., 2021). Previously, we demonstrated that HET enhances gluconeogenesis and thermogenesis by promoting amino acid utility, which is related to the activation of hepatic autophagy and mitophagy, in aged mice with excessively restricted amount of daily food intake as a model of malnutrition (Nahata et al., 2021). However, the underlying molecular mechanism of the energy metabolism is still poorly understood.

In this study, using a food restricted (FR) mouse, we aimed to evaluate the effect of aging as well as that of HET treatment on locomotor activity and liver metabolic function. Further, we also investigated the mechanism that regulates liver metabolic function as well as the involvement of hepatic mitochondria during aging.

## Materials and Methods

### Animals

For our experiments, male C57BL/6J mice were purchased from Charles River Laboratories (Tokyo, Japan). The mice were either 9-week-old (young) or 23–26-month-old (aged). All animals were singly housed in cages in a room with environmentally controlled ambient temperature (23 ± 3^°^C), humidity (50 ± 20%), and lighting (12 h light/dark cycle) conditions. The animals were provided water and a standard laboratory animal diet (MF; Oriental Yeast Co., Ltd., Tokyo, Japan) *ad libitum*. Further, the study was approved by the experimental animal ethics committee at Tsumura & Co. (Tokyo, Japan; permit no. 15-053, 15-074, 16-007, 17-025, 17-036, 17-081, and 18-042), and all the experimental procedures were performed in accordance with ARRIVE guidelines and the guidelines of the National Institutes of Health for the care and use of laboratory animals.

### Test Substance

The traditional Japanese medicine, HET is a powdered hot-water-extract consisting of the following 10 components: Astragalus Root (Astragali radix), 4.0 g; *Atractylodes lancea* Rhizome (Atractylodis lanceae rhizoma), 4.0 g; Ginseng (Ginseng radix), 4.0 g; Japanese *Angelica* root (Angelicae radix), 3.0 g; *Bupleurum* Root (Bupleuri radix), 2.0 g; Jujube (Zizyphi fructus), 2.0 g; *Citrus unshiu* Peel (Aurantii nobilis pericarpium), 2.0 g; *Glycyrrhiza* (Glycyrrhizae radix), 1.5 g; Cimicifuga Rhizome (Cimicifugae rhizoma), 1.0 g; and Ginger (Zingiberis rhizoma), 0.5 g. It was manufactured by Tsumura & Co. (Tokyo, Japan). Supplementary Figure 1 shows the different ingredients in HET, which were identified via three-dimensional high-pressure liquid chromatography.

### Experimental Procedure

Young and aged mice were assigned to either an *ad libitum* fed (Fed) or food-restricted (FR) group. The FR groups were provided with 30% *ad libitum* intake. The feeding regimens were administered for 5 days after which the mice were sacrificed under isoflurane anesthesia, and their liver samples were collected. To examine the effect of HET, aged mice received HET (1.5 wt/wt%)-containing or control pellet chow diet *ad libitum* for 4 weeks, followed by 5 days of FR. During this FR period, HET was mixed with the powdered chow at 50 mg/mouse/day, which was calculated from the amount of HET ingested during *ad libitum* intake. Thus, the aged mice were administered an approximately equal dose of HET daily throughout the experimental period. According to our calculation, we assumed that amino acid content was almost comparable between the control diet and HET-containing diet during food restriction and that the effect of HET was not attributable to amino acid supplementation.

Further, the mice that displayed remarkable hypothermia or a decline in feeding behavior during food restriction were euthanized for ethical reasons. Furthermore, aged mice that developed spontaneous tumors were excluded from the experiment to avoid potential confounding factors.

### Plasma Analysis

To collect blood samples from the abdominal inferior vena cava, the mice were sacrificed under isoflurane anesthesia after 5-day FR. Thereafter, at a temperature of 4^°^C, centrifugation was performed at 10,000 × g for 3 min and plasma samples were obtained. This was followed by the measurement of plasma glucose and transthyretin levels using the Glucose CII Test Wako Kit (Wako Pure Chemical Industries, Tokyo, Japan) and Mouse Prealbumin ELISA Kit (Immunology Consultants laboratory, Portland, OR, United States), respectively. Further, to determine ketone body β-hydroxybutyrate levels using a FreeStyle Precision Neo device (Abbott, Tokyo, Japan), blood samples were collected from the tail vein just prior to feeding on days 1 and 5.

### Liver Metabolomics

The levels of hepatic hydrophilic metabolites were determined via liquid chromatography–tandem mass spectrometry (LC-MS/MS) and the collected data were analyzed using Method Package v2 (Shimadzu, Kyoto, Japan), which contains a mass spectra library and method files specifying analytical conditions, as well as data analysis parameters. The analysis was performed as previously described (Iwasawa et al., 2021).

### Transmission Electron Microscopy

Liver tissues were fixed in 2.5% glutaraldehyde at 4^°^C for 24 h and post-fixed using 1% osmium tetroxide for 1 h. Thereafter, they were dehydrated in alcohol and embedded in epoxy resin, after which ultrathin sections of the liver samples were stained with uranyl acetate and lead citrate, and the mitochondrial morphology of the hepatocytes was analyzed using transmission electron microscope (Hitachi H-7650H, Tokyo, Japan). Further, the mean cross-sectional areas of the mitochondria were assessed using ImageJ software.

### Gene Expression

RNA was extracted from liver samples using the RNeasy MiniKit (Qiagen, Valencia, CA, United States) and reverse transcribed to cDNA using the TaqMan High-Capacity cDNA Reverse Transcription Kit (Thermo Fisher Scientific, Waltham, MA, United States). Thereafter, mRNA expression was analyzed via quantitative real-time polymerase chain reaction (TaqMan Fast Advanced Master Mix, Thermo Fisher Scientific, Waltham, MA, United States) using TaqMan gene-specific primers (Thermo Fisher Scientific, Waltham, MA, United States) on a QuantStudio 7 Flex Real-Time PCR System (Thermo Fisher Scientific, Waltham, MA, United States), and normalized to that of the 18S rRNA gene. The mitochondrial DNA (mtDNA) copy number was calculated using a mitochondrial gene, cytochrome b (Cytb), normalized against a nuclear gene, Rpph1 (Supplementary Table 1).

### Locomotor Activity

Locomotor activity was assessed using an infrared ray sensor (NS-AS01; Neuroscience, Tokyo, Japan). After the 5-day FR, HET-treated aged mice were intraperitoneally injected with L-alanine solution (2 g/kg; Fujifilm Wako Pure Chemical, Osaka, Japan) or saline, and the locomotor activity of the mice was monitored within 30–120 min after the injection. Blood samples were collected from the tail vein immediately prior to injection to determine glucose levels using the OneTouch UltraVue kit (LifeScan, Tokyo, Japan). Mice with hypoglycemia, which was defined as a blood glucose level below 50 mg/dL, were excluded from further analysis for ethical reasons.

### Statistical Analyses

Statistical analysis was performed using GraphPad Prism version 8 (GraphPad Software, San Diego, CA, United States) and StatLight 2000 (Yukms, Tokyo, Japan), and the results presented as the mean ± standard error and as individual data points. Specifically, the results were assessed by performing Tukey-Kramer, Steel–Dwass, or Dunnett’s tests for multiple-group comparisons, and Student’s or the Aspin–Welch t-test for two-group comparisons. Two-way analysis of variance followed by the Bonferroni *post-hoc* test was used for time-course analyses. Further, metabolomics data were processed using R software, and Mann-Whitney *U*-test was used to determine statistically significant changes. *P*-values < 0.05 were considered statistically significant.

## Results

### Biochemical Features

**FIGURE 1:**
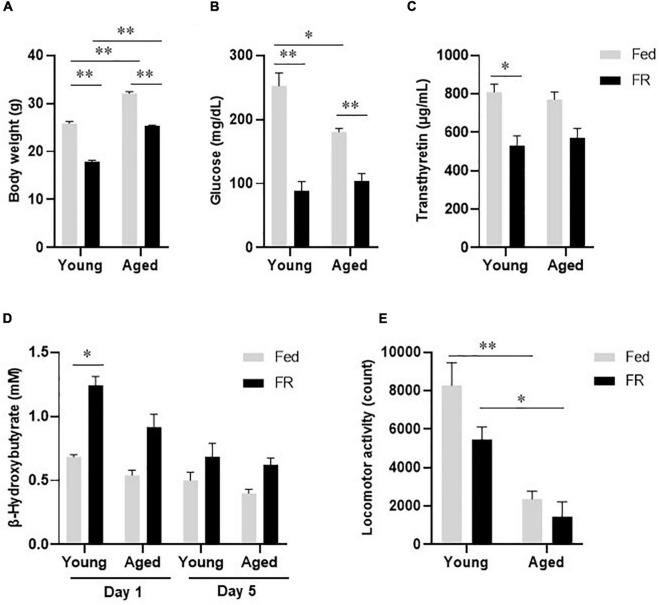
Biochemical and physiological responses to food restriction in young and aged mice. **(A)** Body weight, **(B)** Plasma glucose levels, and **(C)** Plasma transthyretin levels in young and aged mice fed *ad libitum* (Fed) or food restricted (FR). **(D)** Blood β-hydroxybutyrate levels in young and aged mice, on days 1 and 5 after FR. Young Fed, *n* = 6; aged Fed, *n* = 5; young FR, *n* = 7–8; and aged FR, *n* = 11–18. **(E)** Locomotor activity during the dark phase in young and aged mice after FR for 5 days (n = 6 in each group). Data are presented as mean ± standard error (SE). *p < 0.05, ^**^p < 0.01, Two-way analysis of variance (ANOVA) followed by Tukey-Kramer test.

At baseline, the body weights of aged mice were higher than those of young mice, and FR for 5 days resulted in body weight decreases in both mouse groups (Figure 1A). There was no significant difference between young and aged mice in body weight change under FR. FR also resulted in decreases in the plasma glucose (Figure 1B) and transthyretin (Figure 1C) levels in both mouse groups. Further, unlike the aged mice, the young mice showed a significant increase in blood β-hydroxybutyrate level on day 1 after FR. However, on day 5, the two groups showed no significant differences (Figure 1D). Taken together, these results indicated that both young and aged mice became malnourished after 5 days of FR.

### Locomotor Activity

During the dark phase, locomotor activity, which decreased following FR, was significantly lower in aged mice than in young mice (Figure 1E). Two-way analysis of variance revealed no statistically significant interaction between the effects of age and FR [*F*_(1,20)_ = 1.368, *p* = 0.256]. However, the differences in the effects of FR [*F*_(1,20)_ = 5.274, *p* < 0.05] and age [*F*_(1,20)_ = 37.82, *p* < 0.001] on locomotor activity were statistically significant, suggesting a defective energy production system in aged FR mice.

### Metabolomic Profile

**FIGURE 2:**
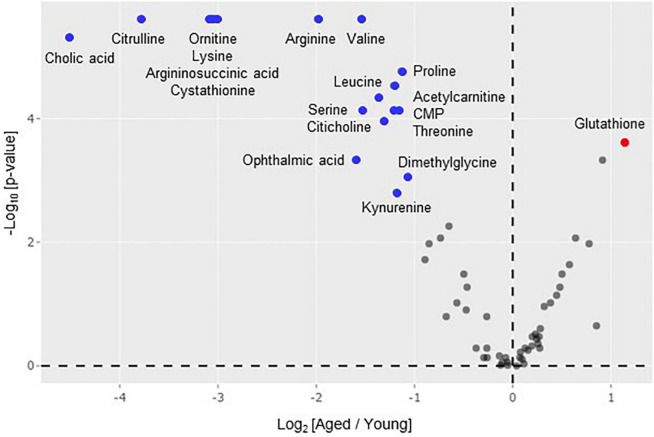
Volcano plot analysis of hepatic metabolome. Differences in metabolite levels (fold change) and statistical significance (*p*-value) between aged (*n* = 9) and young (*n* = 14) mice under food restriction are displayed. The colored points indicate metabolites with a minimum log_2_ fold change of ±1 in aged mice compared with young mice. Metabolomics data were processed using R software and Mann-Whitney *U*-test was used for statistical analysis.

To elucidate the metabolite profiles of the mice, liver samples from both young and aged FR mice were subjected to metabolomic analysis. The 64 metabolites detected in the liver were displayed in a volcano plot, which shows the log_2_ fold-change value (x-axis) versus -log_10_ *p*-value (y-axis) of metabolites in aged FR mice compared with young FR mice (Figure 2). The levels of 18 metabolites (blue dots) were decreased and the level of 1 metabolite (red dot) was increased with a minimum log_2_ fold change of ±1 and a statistical significance of *p* < 0.05. These metabolites were amino acids, including branched-chain amino acids (BCAAs), cystathionine, glutathione (GSH), and metabolites related to urea cycle, as well as carnitine, bile acid, choline, and kynurenine.

**FIGURE 3:**
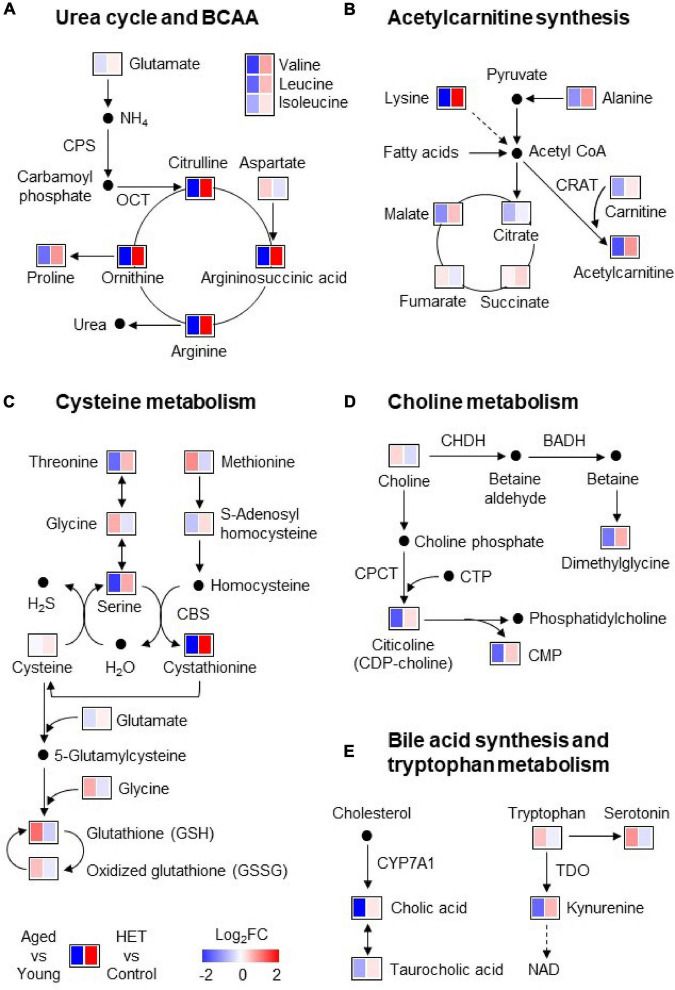
Pathway maps of the metabolic profile of mouse liver under conditions of food restriction. **(A–E)** Metabolic pathways involved in the liver metabolites significantly altered in aged mice. Heatmap showing the fold-change in the level of each metabolite in the liver of aged mice (*n* = 9) (compared with young mice; *n* = 14) and hochuekkito (HET)-treated aged mice (*n* = 10) (compared with control aged mice; *n* = 9). The red and blue regions indicate increases and decreases in the metabolite levels, respectively. BADH, betaine aldehyde dehydrogenase; CBS, cystathionine β-synthase; CHDH, choline dehydrogenase; CMP, cytidine monophosphate; CPCT, CTP:choline-phosphate cytidylyltransferase; CPS, carbamoyl phosphate synthetase-1; CRAT, carnitine acetyltransferase; CTP, cytidine triphosphate; CYP7A1, cholesterol 7α-hydroxylase; NAD, nicotinamide adenine dinucleotide; OCT, ornithine carbamoyltransferase; TDO, tryptophan 2,3-dioxygenase.

Figure 3 shows pathway maps of these metabolites, which are referenced in the Kyoto Encyclopedia of Genes and Genomes (KEGG) pathway database. The fold-change in the level of each metabolite was represented using a Heatmap in the range of ±2. The left and right panels indicate the levels in aged mice compared with those in young mice and HET-treated aged mice compared with those in control aged mice, respectively. The red and blue regions indicate increases and decreases in the metabolite levels, respectively. The pathway analysis revealed that aged FR mice exhibited decreased urea cycle, acetylcarnitine synthesis, cysteine metabolism, choline metabolism, bile acid synthesis, and tryptophan metabolism in the liver (Figure 3). These results suggested that several liver metabolic functions were attenuated in aged FR mice.

**FIGURE 4:**
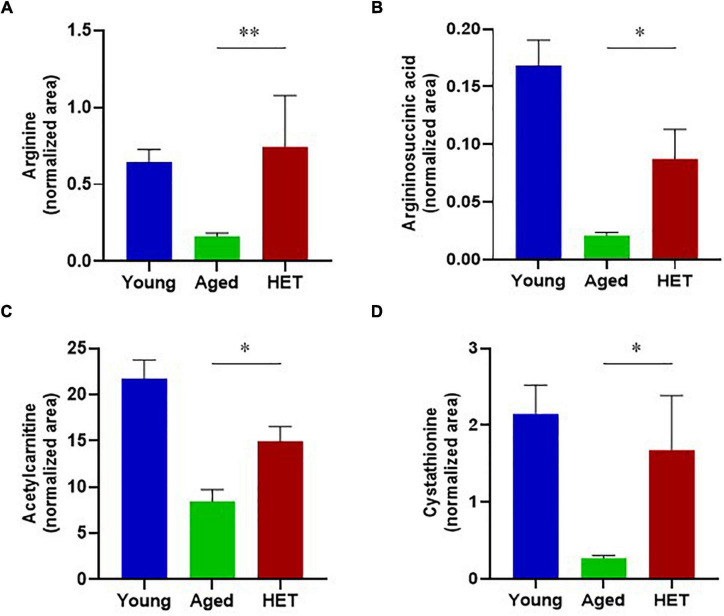
Metabolites that exhibited a significant increase in their levels in hochuekkito (HET)-treated aged mice with food restriction. **(A)** Arginine, **(B)** Argininosuccinic acid, **(C)** Acetylcarnitine, **(D)** Cystathionine. These liver metabolite levels in young mice, aged mice (non-treated controls), and HET-treated aged mice (HET) are represented as peak area values normalized using an internal standard in the liquid chromatography–tandem mass spectrometry (LC-MS/MS) analysis. Young mice, *n* = 14; control aged mice, *n* = 9; HET-treated aged mice, *n* = 10. Data are presented as mean ± standard error (SE). *p < 0.05, ^**^p < 0.01, Mann-Whitney *U*-test.

However, the levels of these metabolites tended to be reversed by HET treatment (Figure 3). Figure 4 shows significantly increased metabolites in HET-treated aged FR mice. The levels in young FR mice, aged FR mice (non-treated controls), and HET-treated aged FR mice were represented as peak area values normalized using an internal standard in the LC-MS/MS analysis. HET treatment resulted in a significant increase in the levels of urea cycle-related metabolites, acetylcarnitine, and cystathionine.

### Mitochondrial Morphology

**FIGURE 5:**
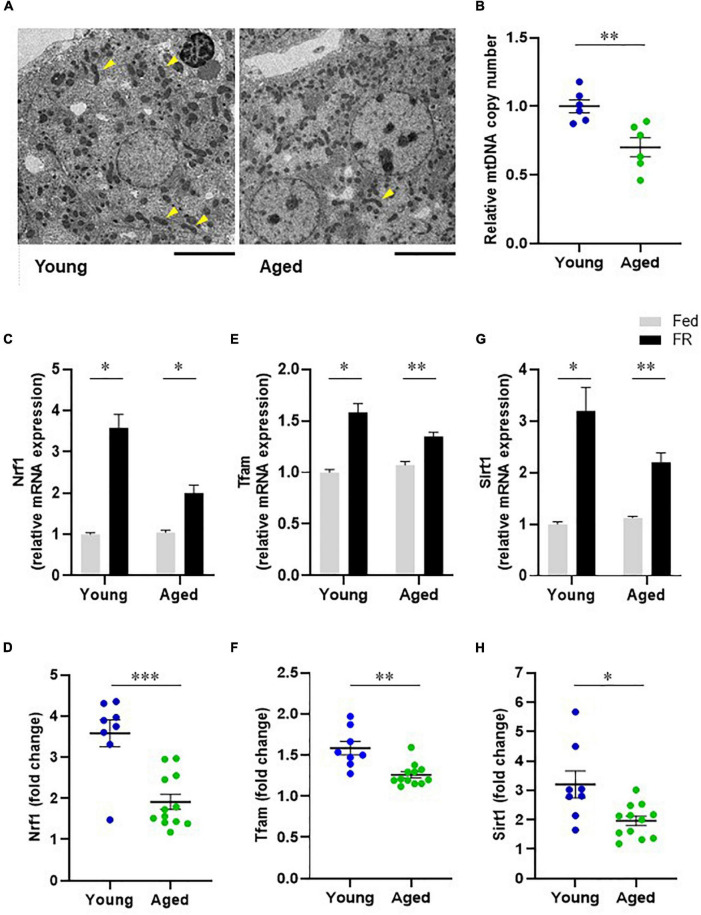
Hepatic mitochondrial biogenesis in young and aged mice. **(A)** Transmission electron microscope images of mitochondria in hepatocytes (Scale bar = 10 μm). Arrowheads show elongated mitochondria. **(B)** Comparison of hepatic mitochondrial DNA (mtDNA) copy numbers corresponding to young and aged mice (*n* = 6 in each group). **(C–F)** mRNA expression levels of mitochondrial biogenesis regulators, nuclear respiratory factor 1 [Nrf1, **(C)**] and mitochondrial transcription factor A [Tfam, **(E)**] in mice with food restriction (FR) compared with mice fed *ad libitum* (Fed). Panels **(D,F)** indicate fold changes in response to FR. **(G)** Sirtuin1 (SIRT1) mRNA expression in both fed and FR mice. **(H)** Change in SIRT1 expression level in response to FR. Young Fed, *n* = 6; aged Fed, *n* = 5; young FR, *n* = 8; and aged FR, *n* = 12. Data are presented as mean ± standard error (SE). *p < 0.05, **p < 0.01, ***p < 0.001; Steel–Dwass tests, multiple-group comparisons; Aspin–Welch *t*-test, two-group comparisons.

To investigate the mitochondrial dynamics that induce changes in liver metabolic function, we compared the hepatic mitochondrial morphologies of young and aged mice. The transmission electron microscopy (TEM) images of mouse hepatocyte mitochondria (Figure 5A) showed elongated mitochondria (arrowheads) for hepatocytes from young mice, while those in hepatocytes from aged mice appeared to be short and globular, with a small volume, suggesting mitochondrial morphological alterations in aged mice. Further, the relative mtDNA copy number in liver samples from aged mice was significantly lower than that from young mice (Figure 5B).

### Sirtuin 1 and Mitochondrial Biogenesis

The mRNA expression levels of mitochondrial biogenesis regulators were assessed to compare the hepatic mitochondrial biogenesis abilities of the young and aged mice. FR markedly elevated the mRNA levels of hepatic nuclear respiratory factor 1 (NRF1) in young mice. However, this response was attenuated in aged mice (Figures 5C,D). Similarly, the mRNA levels of hepatic mitochondrial transcription factor A (TFAM) following FR were significantly lower in aged mice than in young mice (Figures 5E,F). These results could indicate that the hepatic mitochondrial biogenesis ability in aged mice was lower than that in young mice. Further, to elucidate the mechanism underlying the dysregulated hepatic mitochondrial biogenesis in aged mice, we evaluated Sirtuin 1 (SIRT1) expression. The results thus obtained indicated that in young mice, FR upregulated the expression level of SIRT1 mRNA (Figure 5G). Conversely, the induction of SIRT1 expression was suppressed in aged mice compared with that in young mice (Figure 5H), suggesting the involvement of SIRT1 in the downregulation of mitochondrial biogenesis in aged mice.

### Therapeutic Effect of Hochuekkito

**FIGURE 6:**
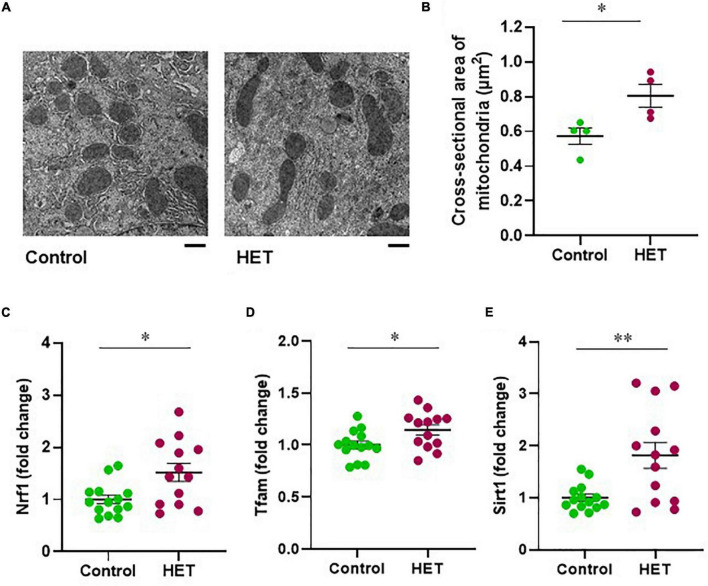
Therapeutic effect of hochuekkito (HET) in aged mice with food restriction. **(A)** Transmission electron microscope images of mitochondria in hepatocytes (Scale bar = 1 μm). **(B)** Mean cross-sectional area of mitochondria in aged mice with or without HET treatment (*n* = 4 in each group). Data are presented as mean ± standard error (SE). *p < 0.05, Student’s *t*-test. **(C–E)** Changes in hepatic nuclear respiratory factor 1 (NRF1), mitochondrial transcription factor A (TFAM), and sirtuin1 (SIRT1) mRNA expression in response to food restriction. Control, *n* = 14; HET-treated, *n* = 13. Data are presented as mean ± standard error (SE). *p < 0.05, **p < 0.01, Aspin–Welch *t*-test.

Next, we evaluated the effects of HET on mitochondrial morphology and biogenesis regulators in aged mice. The mean cross-sectional area of mitochondria in hepatocytes from the livers of HET-treated aged mice was larger than that corresponding to the aged control mice (Figures 6A,B). Further, the mRNA expression levels of NRF1 and TFAM after FR were significantly higher in the HET-treated group than in the control group (Figures 6C,D). Additionally, increased SIRT1 mRNA expression was observed in the HET group (Figure 6E). These results, combined with the findings described above, indicated that HET can restore hepatic mitochondrial biogenesis by inducing SIRT1 expression in aged mice.

**FIGURE 7:**
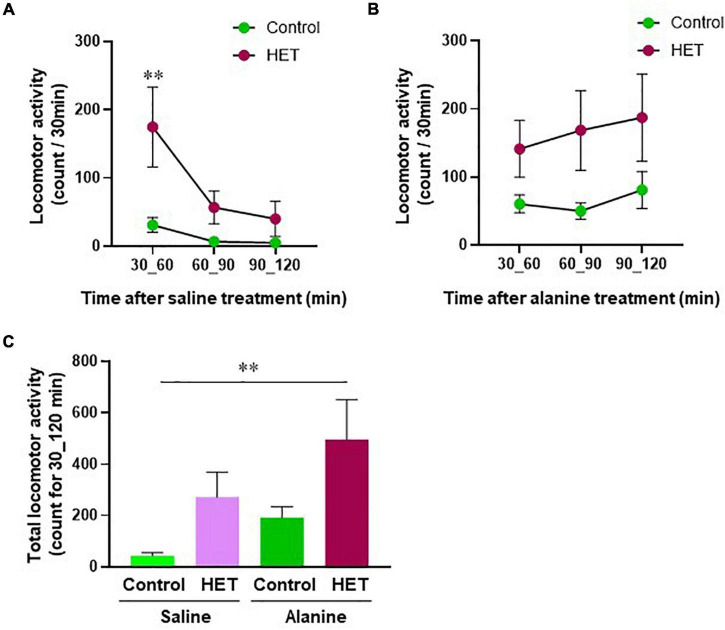
Locomotor activity in aged mice with food restriction. The time-dependent change in the locomotor activity after: **(A)** Saline or **(B)** Alanine injection in the HET-treated aged mouse group and the control. **(C)** Total locomotor activity within 30–120 min after saline or alanine injection. Control/Saline, *n* = 21; HET/Saline, *n* = 25; Control/Alanine, *n* = 25; and HET/Alanine, *n* = 26. Data are presented as mean ± standard error (SE). **p < 0.01, Two-way analysis of variance (ANOVA) followed by the Bonferroni *post-hoc* test, and Dunnett’s test.

We also analyzed the effects of HET on locomotor behavior in association with hepatic energy metabolism. Figures 7A,B show time-dependent changes in locomotor activity in aged FR mice after saline or alanine injection. In particular, locomotor activity after saline injection was significantly higher in the HET-treated group than in the control group (Figure 7A). Further, even though no significant changes were observed after alanine injection, a tendency of increased locomotor activity was observed in the HET-treated aged mouse group (Figure 7B). Total locomotor activity within 30–120 min also showed a significant increase following the combined administration of HET and alanine in aged FR mice compared with that observed after saline-only treatment (Figure 7C).

## Discussion

In this study, we observed that FR resulted in decreased locomotor activity in aged mice as well as changes in liver metabolite profiles. These effects were found to be associated with a decline in the expression levels of SIRT1 and hepatic mitochondrial biogenesis regulators. However, they could be partially reversed by HET treatment.

The liver, as a central metabolic organ, plays an essential role in maintaining whole-body homeostasis via the regulation of systemic energy metabolism (Hunt et al., 2019). Under fasting conditions, it produces endogenous glucose from glycogen or amino acids and generates ketone bodies via hepatic mitochondrial β-oxidation to provide energy for extrahepatic tissues (Rui, 2014). Accordingly, it is considered that age-related metabolic dysfunction in the liver contributes to a decline in whole-body functions. In this study, we observed that the increase in blood β-hydroxybutyrate level on day 1 after FR treatment was attenuated in aged mice compared with young mice. Moreover, metabolomic analysis revealed that aged FR mice exhibited reduced levels of hepatic metabolites, such as amino acids, including BCAAs, carnitine, cystathionine, and the metabolites related to the urea cycle, as well as bile acid, choline, and kynurenine synthesis. Approximately 30% of liver metabolites were significantly decreased in aged FR mice compared with those in young FR mice. These findings suggest that aged mice are more vulnerable to metabolic stress. Metabolic pathway analysis revealed that the urea cycle, acetylcarnitine synthesis, cysteine metabolism, choline metabolism, bile acid synthesis, and tryptophan metabolism were decreased in the liver of aged FR mice. It has been reported that several of these pathways are associated with mitochondrial function (Cohen et al., 1992; Zhang et al., 1992; Pietruszko and Chern, 2001; Pesce et al., 2012; Vicente et al., 2016; Mezhnina et al., 2020).

The urea cycle in the liver is the main metabolic pathway by which ammonia is converted to urea. The enzymes that play a key role in this process are carbamoyl phosphate synthetase-1, which produces carbamoyl phosphate from ammonia, and ornithine carbamoyltransferase, which catalyzes the formation of citrulline from carbamoyl phosphate and ornithine (McGivan et al., 1976). Further, these enzymes are localized in the matrix of the hepatocyte mitochondria of ureotelic animals (Cohen et al., 1992). During fasting, the urea cycle is induced by stimulating amino-acid oxidation or gluconeogenesis in the liver (Sokolović et al., 2008). Therefore, the decreased urea cycle metabolites in aged FR mice suggest a decline in the clearance of ammonia generated from increased amino acid catabolism. It has also been reported that urea cycle disorders result in decreases in the levels of BCAAs, such as leucine, isoleucine, and valine, owing to the enhanced conversion of BCAAs to glutamine to the end of detoxifying ammonia in muscles (Holecek, 2018). Therefore, the decreased liver BCAA levels observed in this study could be attributed to the metabolic dysregulation of the urea cycle in aged FR mice. Additionally, the release of acetylcarnitine, which is a metabolite generated from acetyl-CoA and carnitine by mitochondrial carnitine acetyltransferase, is induced in calorie restriction and helps to control energy metabolism (Mezhnina et al., 2020). Acetylcarnitine treatment reportedly prevents age-related reduction in liver mitochondrial biogenesis (Pesce et al., 2012). Furthermore, cystathionine is generated from the reaction between homocysteine and serine or cysteine, which is catalyzed by cystathionine β-synthase (CBS) and results in hydrogen sulfide production (Singh et al., 2009). CBS is a cytosolic enzyme; however, it can be partially localized in liver mitochondria to offer protection against oxidative damage (Vicente et al., 2016), and reportedly, plasma hydrogen sulfide levels as well as CBS expression in the kidney decrease during aging (Hou et al., 2016). Thus, hydrogen sulfide is involved in aging and age-related diseases (Zhang et al., 2013). In conclusion, this suggests that the changes in the liver metabolic profile could indicate a dysregulation of mitochondrial metabolic function in aged FR mice.

Meanwhile, liver tryptophan 2,3-dioxygenase, an intracellular cytosolic enzyme, converts tryptophan to kynurenine, leading to *de novo* nicotinamide adenine dinucleotide [NAD (+)] synthesis (Castro-Portuguez and Sutphin, 2020). NAD (+) is an essential cofactor needed for many metabolic reactions and mitochondrial energy production (Katsyuba et al., 2018). Reportedly, tryptophan 2,3-dioxygenase activity and NAD levels decrease during aging (Comai et al., 2005). Accordingly, these metabolic changes are not directly associated with decreased metabolic function in liver mitochondria; however, they may influence the regulation of energy homeostasis in aged FR mice.

Glutathione is primarily a mitochondrial oxidative stress marker (Maher, 2005). Previous studies demonstrated a significant reduction in its levels in several tissues of aged mice with constant food supply (Maher, 2005). However, after overnight fasting, the GSH levels were higher in the 103-weeks-old mice than in the 24-weeks-old mice (Houtkooper et al., 2011). This observation corroborates the findings in this study. Nutrient deprivation markedly decreases both the reduced GSH and the oxidized GSH in the liver (He et al., 2015). Moreover, a starvation-induced decline in GSH levels depends on the removal of GSH from the intracellular environment, and this is promoted by autophagy (Desideri et al., 2012). Our previous research demonstrated a reduced hepatic autophagy in aged FR mice (Nahata et al., 2021), suggesting a precise correlation of autophagy with increased GSH levels.

The conversion of choline to dimethylglycine is mediated by the liver mitochondrial enzymes choline dehydrogenase (Zhang et al., 1992) and betaine aldehyde dehydrogenase (Pietruszko and Chern, 2001). Citicoline is an intermediate metabolite during the synthesis of phosphatidylcholine from choline; this results in the conversion of CTP to CMP, a reaction that is catalyzed by CTP: choline-phosphate cytidylyltransferase (CPCT), which is localized in the cytosol and microsomes of the liver and brain (Weiss, 1995). During aging, CPCT activity is reduced in the brain, but the same is not observed in the liver (Lepagnol and Heidet, 1993). However, in this study, the level of liver citicoline during FR was found to be lower in aged mice than in young mice, but the causal mechanism is unclear.

In this study, we observed that the level of cholic acid and the ratio of cholic acid to taurocholic acid in the liver during FR were decreased significantly in aged mice compared to those in young mice. This observation is consistent with the previously reported findings of several studies (Einarsson et al., 1985; Lee et al., 2016; Ma et al., 2020; Han et al., 2021), signifying that these changes are the characteristic features of the aging liver. Bile acids, including cholic acid and taurocholic acid, are essential for the absorption of lipophilic molecules, such as lipids and fat-soluble vitamins, contributing to the maintenance of gut microbiota, and lipid, glucose, and energy homeostasis by activating farnesoid X receptor and Takeda G protein–coupled receptor 5 (Chiang and Ferrell, 2019). Notably, accumulating evidence suggests that bile acids play a pivotal role in enhancing longevity in animals (Zhou et al., 2021).

Hochuekkito is used to treat certain clinical conditions, such as physical function failure and general weakness in elderly patients with malnutrition, chronic obstructive pulmonary disease, or infection (Kuroiwa et al., 2004; Satoh et al., 2005; Hamada et al., 2018; Akita et al., 2019; Kohno et al., 2021). In this study, we observed that the change in the metabolite profile of the liver of aged FR mice was partially restored following HET treatment. These findings suggest that HET ameliorates metabolic dysfunctions of the liver in aged mice and modulates the energy homeostasis of the entire body. Moreover, the decline in the levels of the urea cycle metabolites was effectively reversed by HET treatment, implying that HET facilitates the clearance of ammonia under the FR conditions. We speculate that these effects of HET on the liver metabolite profiles may be attributed to the metabolic function of hepatic mitochondria.

During the aging process, the decrease in mitochondrial DNA volume and the accumulation of oxidative damage are induced by reactive oxygen species generation (Chistiakov et al., 2014). Further, the mitochondrial quality control system involved in mitochondrial maintenance can be dysregulated during aging, and this critically influences whole-body metabolism, health, and lifespan in the elderly (Davinelli et al., 2020). In this study, a decrease in the relative mtDNA copy number as an index of cellular mitochondrial content was observed in the livers of aged mice, suggesting hepatic mitochondrial bioenergetics dysfunction.

Mitochondrial biogenesis increases the capacity for oxidative phosphorylation, decreases oxidative stress, and alleviates mitochondrial dysfunction (Popov, 2020). Reportedly, it is induced by environmental stressors, such as caloric restriction, endurance exercise, oxidative stress, and malnutrition to the end of maintaining bioenergetic efficiency (López-Lluch et al., 2006; Nirwane and Majumdar, 2018). Our previous report demonstrated a decline in peroxisome proliferator-activated receptor co-activator 1 alpha (PGC-1α) in aged FR mice compared to young FR mice (Nahata et al., 2021). In particular, PGC-1α is a key sensor of mitochondrial biogenesis and regulates the downstream transcription factors, NRF1 and TFAM (Popov, 2020). In this study, the mRNA expression levels of NRF1 and TFAM were increased by FR in young mice, while these changes were attenuated in aged mice. These findings indicate a decline in hepatic mitochondrial biogenesis function during aging that could be related to the decreased metabolic function of aged livers.

Sirtuin 1, a member of the sirtuin family of NAD (+)-dependent deacetylases, is intimately associated with cellular metabolism. It is activated by a sense of energy deprivation and also promotes several metabolic functions in the liver, including gluconeogenesis via PGC-1α and fatty acid oxidation, to the end of protecting the whole body against metabolic stresses (Chang and Guarente, 2014). Moreover, SIRT1 functionally interacts with several transcriptional factors, resulting in enhanced mitochondrial biogenesis (Nisoli et al., 2005; Yuan et al., 2016); thus, it plays an important role in metabolic health, implying that the decline in the metabolic function of the liver of aged FR mice was possibly mediated by the suppressed induction of hepatic SIRT1 expression.

**FIGURE 8:**
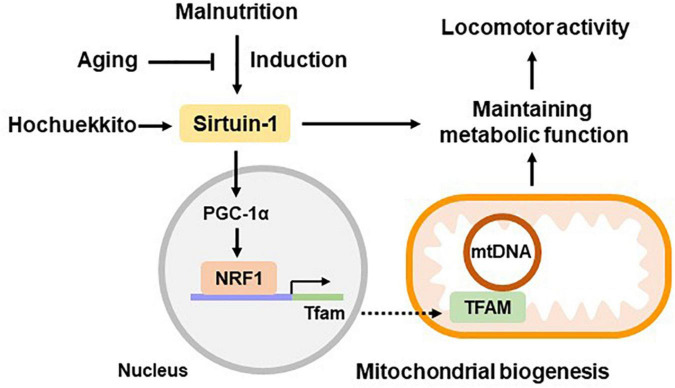
Hypothetical scheme of liver metabolic function during aging. Hepatic sirtuin1 (SIRT1) expression was induced by malnutrition and regulated metabolic functions by partially promoting the expression of mitochondrial biogenesis regulators, peroxisome proliferator-activated receptor co-activator 1 alpha (PGC-1α), nuclear respiratory factor 1 (NRF1), and mitochondrial transcription factor A (TFAM). These signaling pathways showed decline during the aging process, resulting in decreased energy production. Hochuekkito showed efficacy in ameliorating hepatic metabolic function and locomotor activity, which is mediated via SIRT1 upregulation. Data related to PGC-1α were based on our previous study using the same model as that in this study (Nahata et al., 2021).

Additionally, HET treatment ameliorated the FR-induced decrease in SIRT1 gene expression in the livers of aged mice. It also increased hepatic mitochondrial volume and promoted FR-induced NRF1 and TFAM expression in aged mice. We previously reported that liver PGC-1α was restored by HET treatment in a study using the same model as that in this study, and HET promoted the activation of hepatic autophagy (Nahata et al., 2021). Autophagy is known to be activated through the SIRT1 signaling pathway (Salminen and Kaarniranta, 2009; Ng and Tang, 2013). Therefore, the effect of HET on metabolic function in hepatic mitochondria could be mediated by activating SIRT1, which is partially involved in the upregulation of liver mitochondrial biogenesis (Figure 8).

Locomotor function can be closely related to muscle mitochondrial impairment. However, recent studies demonstrate the importance of liver-muscle crosstalk in sarcopenia and physical frailty. Metabolic abnormalities, such as hypoglycemia, hyperammonemia, and BCAAs deprivation alter energy and protein homeostasis of the whole body, including skeletal muscle (Holeček et al., 2011; Qiu et al., 2013; Davuluri et al., 2016; Ebadi et al., 2019; Allen et al., 2021). Our previous report showed that food restriction-induced liver gluconeogenesis depending on hepatic autophagy was associated with spontaneous locomotor activity in mice (Nahata et al., 2021). Liver gluconeogenesis is controlled by SIRT1 and mitochondria (Rodgers et al., 2005; Rui, 2014). Accordingly, we analyzed locomotor activity, which is closely associated with energy metabolism. Our results revealed that aged FR mice showed lower locomotor activity than young FR mice. They also indicated that in aged FR mice, HET-only treatment significantly enhanced locomotor activity. Further, locomotor activity was increased by the liver glucogenic amino acid alanine supplementation in aged FR mice, which was potentiated by HET treatment. Consequently, these changes in locomotor activity owing to HET treatment may be mediated by the amelioration of the metabolic function of hepatic mitochondria. It has also been reported that HET activates both mitochondrial and glycolytic energy metabolism in cells impaired by influenza A virus infection (Takanashi et al., 2017). This report is consistent with our finding that HET can maintain energy homeostasis.

In this study, we could not determine the major active ingredients of HET; however, it has been reported that some HET ingredients can affect mitochondrial function in animal models. Specifically, astragaloside IV, an ingredient of Astragalus Root extract, enhances lipid metabolism and mitochondrial fatty acid β-oxidation in the liver of aged mice (Luo et al., 2021). Further, ginsenosides, which are the major ingredients of ginseng, regulate mitochondrial energy metabolism and biosynthesis, resulting in the maintenance of mitochondrial function (Wang and Roh, 2020), and atractylodin, an active ingredient of *A. lancea* rhizome, is involved in SIRT1 expression activation (Fujitsuka et al., 2016). Therefore, the therapeutic effects of HET could be partially attributed to these compounds.

Maintaining healthy mitochondria is essential for cell homeostasis; however, age-dependent abnormalities in mitochondrial turnover lead to impaired mitochondrial function (Ploumi et al., 2017). Further, in mitochondrial biogenesis, mitophagy plays a role in degrading dysfunctional mitochondria, thereby regulating mitochondrial quality and participating in energy metabolism modulation (Ploumi et al., 2017). In our previous report, we observed that the decrease in mitophagy marker protein, PTEN induced putative kinase 1 (PINK1) and BCL2 interacting protein 3 (BNIP3) expression in the liver of aged mice was improved by HET treatment (Nahata et al., 2021). Overall, these findings suggest that HET treatment could restore the decline in mitochondrial turnover and repair oxidative stress-induced mitochondria damaged during the aging process. However, further studies are needed in this regard. Additionally, the metabolic profile of the liver of aged FR mice possibly provides valuable information regarding hepatic mitochondria function during aging. Nonetheless, more experiments are needed to validate the results.

In conclusion, aged FR mice showed decreased locomotor activity, which might be related to the metabolic dysfunction of hepatic mitochondria owing to decreased SIRT1 expression during the aging process. Further, HET showed the ability to enhance locomotor activity by restoring these metabolic functions via SIRT1 upregulation. These results provide new insights into the role of HET in elderly patients with malnutrition.

## Data Availability Statement

The original contributions presented in the study are included in the article/Supplementary Material, further inquiries can be directed to the corresponding author.

## Ethics Statement

The animal study was reviewed and approved by the Experimental Animal Ethics Committee at Tsumura & Co.

## Author Contributions

MN, NF, HS, SI, and SM designed and performed the experiments. CS and KO analyzed the metabolic profile. NF and HT conceived and designed the study. MN and NF drafted and revised the manuscript. HT and SO supervised the study. All authors analyzed and interpreted the data and read and approved the final version of the manuscript.

## Conflict of Interest

MN, NF, HS, CS, KO, SI, and SM were employed by Tsumura & Co. HT and SO received grant support from Tsumura & Co.

## Publisher’s Note

All claims expressed in this article are solely those of the authors and do not necessarily represent those of their affiliated organizations, or those of the publisher, the editors and the reviewers. Any product that may be evaluated in this article, or claim that may be made by its manufacturer, is not guaranteed or endorsed by the publisher.
