# Supplementary material for: Decline in Liver Mitochondria Metabolic Function Is Restored by Hochuekkito Through Sirtuin 1 in Aged Mice With Malnutrition
Source: Front Physiol. 2022 Mar 1;13:848960. doi: 10.3389/fphys.2022.848960 (PMC8921682; doi:10.3389/fphys.2022.848960)
Supplement: Supplementary file 1 [file Table_1.DOCX]

Supplementary Table 1. List of gene names used in this study.

Gene names Accession No. Translated products

18S Hs99999901_s1 eukaryotic 18S rRNA

Tfam Mm00447485_m1 transcription factor A, mitochondrial

Nrf1 Mm01135606_m1 nuclear respiratory factor 1

Sirt1 Mm01168521_m1 sirtuin 1

Cytb Mm04225271_g1 cytochrome b

Rpph1 Mm04336066_s1 ribonuclease P RNA component H1
