# Supplementary material for: Decline in Liver Mitochondria Metabolic Function Is Restored by Hochuekkito Through Sirtuin 1 in Aged Mice With Malnutrition
Source: Front Physiol. 2022 Mar 1;13:848960. doi: 10.3389/fphys.2022.848960 (PMC8921682; doi:10.3389/fphys.2022.848960)
Supplement: Supplementary file 2 [file Image_1.pdf]

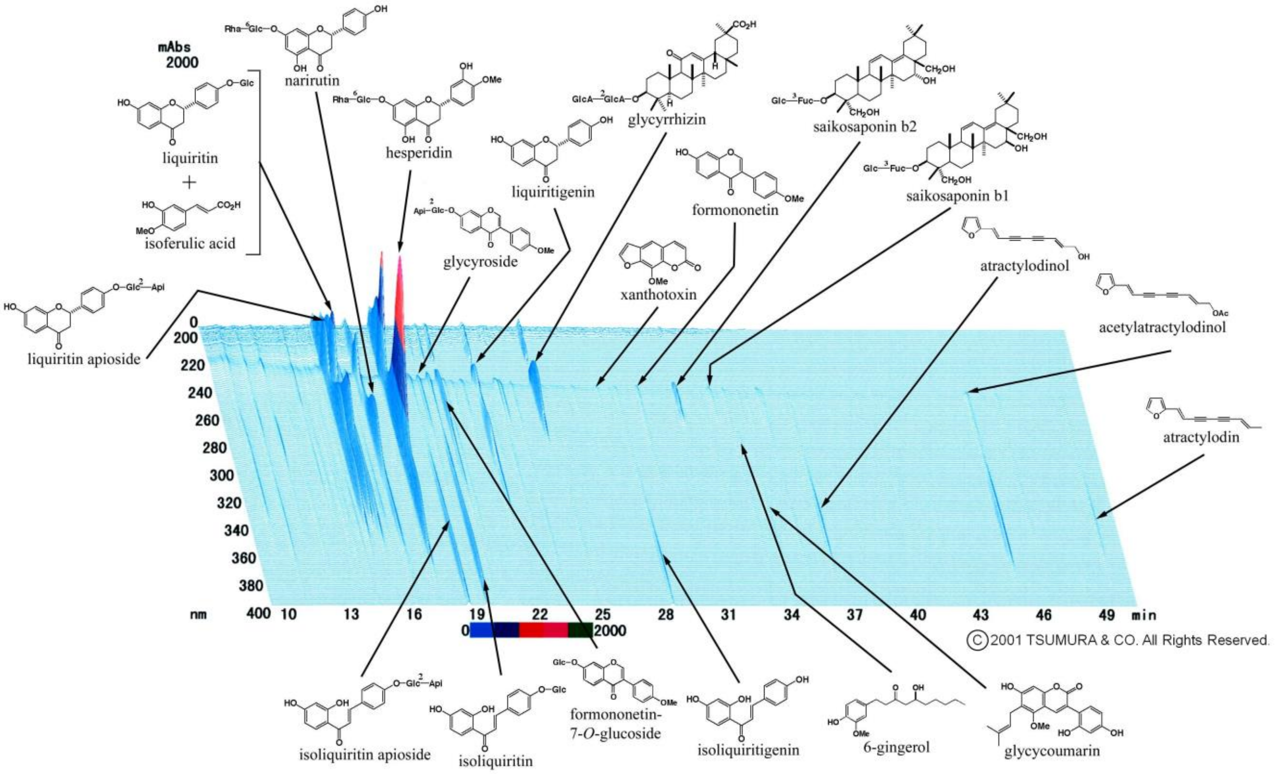

Supplementary Figure 1. A three-dimensional high-performance liquid chromatography profile of hochuekkito, provided by Tsumura & Co., Tokyo, Japan.
